# Assessing differences in emotional intelligence among lifestyle and AI usage profiles generated via Self-Organizing Maps in first-year university students

**DOI:** 10.3389/fpsyg.2026.1905299

**Published:** 2026-08-26

**Authors:** Lucía Alonso-Larza, Rocío Fernández-Piqueras, Adoración-Reyes Moliner Albero, Adrià Marco-Ahulló

**Affiliations:** 1 Department of Psychology, Inclusive Education and Social and Community Development, Catholic University of Valencia “San Vicente Mártir”, Valencia, Spain; 2 Department of General Didactics, Educational Theory and Technological Innovation Catholic University of Valencia “San Vicente Mártir”, Valencia, Spain; 3 Department of Personality, Treatments and Methodology, Faculty of Psychology, Universidad Católica de Valencia “San Vicente Mártir”, Valencia, Spain

**Keywords:** Artificial Intelligence, digital flourishing, emotional intelligence, person-centered approach, university transition

## Abstract

**Background:**

The transition to higher education exposes first-year students to significant lifestyle changes and the challenges of a hyperconnected ecosystem, including the rapid integration of Artificial Intelligence (AI). While physical activity and digital flourishing are recognized as protective factors, their interaction with students' emotional competencies remains underexplored through non-linear, multidimensional approaches.

**Objective:**

This study aimed to identify topological profiles among incoming university students based on lifestyle, digital flourishing, and AI usage, and to analyze differences in intrapersonal emotional intelligence across these distinct profiles.

**Methods:**

A cross-sectional study was conducted with 385 first-year students. Data on physical activity, digital flourishing, AI perceptions and usage, and emotional intelligence were collected using validated self-reported instruments. A person-centered topological approach using Self-Organizing Maps (SOM) and k-means clustering was employed to identify student profiles, followed by one-way ANOVA to evaluate differences in emotional attention, clarity, and repair.

**Results:**

The SOM analysis successfully identified six distinct behavioral and psychosocial profiles. A clear divergence emerged between an adaptive profile (Cluster 6, characterized by active lifestyles, high digital flourishing, and instrumental AI use) and a vulnerable profile (Cluster 1, marked by relative physical inactivity and lower scores across digital dimensions). Crucially, the inferential analysis revealed that the profiles exhibited significant differences in their emotional competencies, with the adaptive profile showing significantly higher levels of emotional clarity and repair compared to the vulnerable group.

**Conclusion:**

Successful adaptation to the university and technological environment transcends the mere acquisition of isolated skills. Intrapersonal emotional intelligence acts as a key psychological factor differentiating adaptive from vulnerable student profiles, underscoring the potential benefit of holistic institutional interventions that simultaneously integrate the promotion of physical activity, digital wellbeing, and emotional competence.

## 1 Introduction

At present, the world is undergoing a revolution driven by the rapid digitalization of recent decades and the recent irruption of Artificial Intelligence (AI) into daily life (Casal-Otero et al., 2023; Hornberger et al., 2023; Kamalov et al., 2023). In this regard, Higher Education has not been immune to this phenomenon. Indeed, it has emerged as one of the key ecosystems where the impact of these tools is driving significant challenges and transformations (Bates et al., 2020; Sun et al., 2026). However, the study of the integration of these technologies within the university setting should not be exclusively limited to their technical dimensions. It is essential to address other intrinsic student factors, such as their perceptions of these tools or the emotional reactions triggered by their use (Ortega-Ochoa et al., 2024; Stöhr et al., 2024; Suh, 2025).

This psychosocial and technological challenge converges with a critical period in the students' development: the transition and adaptation to their first year of university. During this stage, incoming students experience significant changes in their responsibilities and lifestyles (Auerbach et al., 2018; Duffy et al., 2020; Slykerman and Mitchell, 2021), which they must now navigate within an inevitably hyperconnected environment. Within this scenario, the concept of digital flourishing becomes particularly relevant (Janicke-Bowles et al., 2023; Zarco-Alpuente et al., 2026). This multidisciplinary construct transcends the mere use of technology, assessing the students' capacity to exercise self-control, establish meaningful connections, present themselves authentically, and participate civically within virtual environments, thereby achieving a positive impact on their development and wellbeing.

Furthermore, to cope with these academic and digital demands, incoming students require personal resources that act as protective factors. In this regard, students‘ health habits, specifically their physical activity levels, can play a crucial role in maintaining this balance. Scientific evidence warns that the transition to university is often accompanied by an increase in sedentary behavior and a reduction in physical activity levels, driven in part by the new time and social constraints imposed by the university environment (Wilson et al., 2021). However, maintaining an active lifestyle not only provides physiological benefits but is also associated with enhanced psychological variables, such as emotional regulation and self-efficacy, within this population (San Román-Mata et al., 2020; Wang et al., 2025). Nevertheless, recent literature underscores that this protective effect of physical activity may not operate in isolation or unidimensionally. To prevent problematic usage patterns in the face of hyperconnectivity, the benefits of exercise interact synergistically with other psychological resources of the student (Pirwani and Szabo, 2024). Consequently, it is imperative to adopt an integrative approach that not only assesses physical activity levels but also analyzes how these combine with students' emotional competencies to effectively modulate their adaptation to digital environments.

Along these lines, intrapersonal emotional intelligence (understood as the individual's ability to pay attention to, clearly understand, and repair their own affective states) (Fernandez-Berrocal et al., 2004) is posited as an essential transversal competence. Research such as that by Mohamed et al. (2025) suggests that adequate emotional management can mitigate the stress and uncertainty inherent to the university transition. Likewise, studies focused on other populations indicate that emotional intelligence can also facilitate better management of the adverse effects of disruptive technologies (Shaban et al., 2025). Furthermore, recent evidence in higher education shows that student profiles with greater emotional clarity and repair demonstrate a more effective and balanced adoption of automated systems such as AI (Suriá-Martínez et al., 2025). In this way, emotional intelligence is posited as a fundamental psychological factor whose distribution and influence across the various profiles of active lifestyles and student technological adoption require a comprehensive evaluation during this transition period.

Despite the theoretical interconnection among these dimensions, as far as the authors know, much of the prior literature has addressed them in isolation or through linear statistical models that oversimplify a multidimensional reality. To overcome this limitation, the present study adopts a person-centered analytical approach. Consequently, the primary objective of this research was to identify and characterize topological profiles among incoming university students based on their sociodemographic variables, physical activity levels, digital flourishing dimensions, and their patterns of perception, reaction, and use of AI, employing Self-Organizing Maps (SOM). As a secondary objective, and to determine the protective role of affective competencies within these complex groupings, this study aims to analyze whether significant differences exist in the dimensions of emotional intelligence (attention, clarity, and repair) across the distinct profiles emerging from the neural network.

## 2 Materials and methods

### 2.1 Participants

An exploratory, cross-sectional study was conducted to identify topological profiles among first-year university students. A convenience sample of 385 students [298 females, 87 males; 20.2 (4.51) years-old] enrolled in various degree programs at a Spanish university participated in the study. Due to the exploratory nature of the research and the use of unsupervised artificial neural networks (SOM) for pattern recognition, a priori power analysis was not performed. Participation was strictly voluntary, and all individuals provided informed consent.

### 2.2 Procedure

Data collection was carried out during the second semester of the 2025/2026 academic year through a self-administered online survey. The questionnaires were administered online during regular class hours with the prior authorization of the teaching staff. The researchers explained the objectives of the study and guaranteed confidentiality and anonymity of the responses. The average time for completion was approximately 15 min.

Regarding ethical considerations, the study was conducted in accordance with the principles of the Declaration of Helsinki. The protocol was reviewed by the Institutional Ethics Committee of the Catholic University of Valencia, which determined that formal ethical approval was not required, as the research involved a completely anonymous, non-invasive survey with no potential risk to participants. No incentives or rewards were provided for participation.

### 2.3 Instruments

#### 2.3.1 Family Affluence Scale (FAS-III)

The socioeconomic status of the participants was assessed using the third version of the Family Affluence Scale (FAS-III) (Torsheim et al., 2016). In its Spanish version, this instrument has proven to be a valid indicator of household material wealth, having been used in both adolescent and adult populations (Shankar-Krishnan et al., 2018; Chillón et al., 2021).

The questionnaire consists of six closed-ended, self-reported items regarding the availability of material assets and family activities (such as the number of computers, the number of bathrooms in the home, or the frequency of holiday trips). The total sum of the items generates a continuous variable with a score ranging from 0 to 13, where higher values are interpreted as a higher family socioeconomic status.

#### 2.3.2 Student perceptions toward the integration of AI (PEIIA)

To assess students' perceptions toward the incorporation of AI, an adaptation of the PEIIA scale (Perception of AI in Higher Education) was used, which was originally developed for a university population (Torres-Gastelú and Torres-Real, 2025). From the dimensions comprising the original instrument, only two subscales relevant to the research objectives were selected and applied in the present study: Emotional Reactions toward AI (RIA) and AI Accuracy (PIA).

The original instrument uses a five-point Likert-type response format ranging from “strongly disagree” (1) to “strongly agree” (5). The RIA consists of 5 items and evaluates students' affective responses and concerns regarding the technology. This subscale showed adequate internal consistency with a Cronbach's alpha of 0.851. Regarding its construct validity, the Confirmatory Factor Analysis (CFA) yielded optimal goodness-of-fit indices (CFI = 0.996; SRMR = 0.013; RMSEA = 0.041; AGFI = 0.985). On the other hand, the PIA consists of 4 items that analyze trust in the capabilities and the reliability of the results generated by AI. This subscale reported high reliability with a Cronbach's alpha of 0.871. The CFA also supported the model, obtaining acceptable fit indices (CFI = 0.990; SRMR = 0.018; AGFI = 0.950), with the exception of the RMSEA (0.093).

Furthermore, two additional items employing the same response scale were included in this questionnaire to assess the frequency of AI usage and the degree to which it is utilized as a psychological assistant.

#### 2.3.3 Digital Flourishing Scale for Adolescents (DFSA)

To assess the participants' positive digital experiences, the Spanish adaptation of the Digital Flourishing Scale for Adolescents (DFSA) was used (Zarco-Alpuente et al., 2026). This instrument consists of 21 items grouped into five dimensions: connectedness, authentic self-presentation, positive social comparison, civil participation, and self-control. Responses are collected using a 5-point Likert-type scale ranging from 1 (“not at all true of me”) to 5 (“very true of me”), including an additional “not applicable to me” option. In its original validation study within the Spanish population, the CFA supported the model of five correlated factors, obtaining excellent goodness-of-fit indices (CFI = 0.948; TLI = 0.939; RMSEA = 0.037; SRMR = 0.033). Furthermore, the instrument demonstrated strict invariance across age and metric invariance across gender. Regarding its internal consistency, the subscales showed the following reliability values (Cronbach's alpha and McDonald's omega, respectively): authentic self-presentation (0.82; 0.86), self-control (0.79; 0.83), positive social comparison (0.78; 0.81), civil participation (0.73; 0.76), and connectedness (0.65; 0.68).

#### 2.3.4 Trait Meta-Mood Scale (TMMS-24)

Self-perceived emotional intelligence was assessed using the Spanish version of the Trait Meta-Mood Scale (TMMS-24), adapted and validated by Fernandez-Berrocal et al. (2004) from the original scale by Salovey et al. (1995). This instrument consists of 24 items equally grouped into three dimensions that evaluate the intrapersonal aspects of emotional intelligence: Attention to feelings, Emotional clarity and Emotional repair. Responses are recorded on a 5-point Likert-type scale ranging from 1 (“strongly disagree”) to 5 (“strongly agree”). In its original validation study within the Spanish population, the TMMS-24 showed excellent psychometric properties, demonstrating high internal consistency through Cronbach's alpha across its three subscales: Emotional attention (0.90), Emotional clarity (0.90), and Emotional repair (0.86). Regarding its construct validity, recent psychometric studies conducted in Spanish-speaking university populations have supported the three-factor oblique structure of the scale through CFA, reporting adequate goodness-of-fit indices (CFI = 0.938; TLI = 0.931; RMSEA = 0.077) (Gonzalez et al., 2021).

Conceptually, this measure is grounded in the ability model proposed by Mayer and Salovey (1997). From this perspective, emotional intelligence is conceptualized as a set of cognitive skills involved in the identification, processing, and management of emotional information, allowing for the adaptive use of emotions to facilitate thought and behavior (Mayer and Salovey, 1997; Mayer et al., 2000). This framework posits that cognitive and emotional processes are closely intertwined; thus, emotions actively contribute to reasoning, problem-solving, and effective environmental adaptation. Specifically, the model comprises four interrelated branches of emotional information processing: the perception and expression of emotion; the emotional facilitation of thinking (assimilation); the understanding and analysis of emotional states; and the reflective regulation of one's own and others‘ emotions to promote personal growth. While grounded in this overarching ability framework, the TMMS-24 specifically operationalizes the intrapersonal dimensions of the model through a self-report approach, assessing individuals' perceived self-efficacy in attending to, clarifying, and repairing their emotional states.

#### 2.3.5 International Physical Activity Questionnaire-Short Form (IPAQ-SF)

Physical activity levels were estimated using the short form of the International Physical Activity Questionnaire-Short Form (IPAQ-SF). This instrument consists of seven retrospective items that record the frequency (days per week) and duration (minutes per day) participants spent walking, as well as engaging in moderate and vigorous physical activities over the last seven days, additionally including a question about daily sedentary time. Regarding its temporal reliability, international literature has shown that the instrument possesses high test-retest stability after 1 week (*r* = 0.80) (Craig et al., 2003). Regarding its criterion validity for the study population, recent research in Spanish university students has supported the use of the IPAQ-SF by finding positive and significant correlations (*r* = 0.49) with physical activity measurements obtained through triaxial accelerometry (Rodríguez-Muñoz et al., 2017).

### 2.4 Data analysis

The subscale scores for the PEIIA and TMMS-24 were calculated by summing the items that compose them. This same procedure was applied to obtain the global score for FAS-III. However, to calculate the DFSA subscales, it was necessary to compute the mean score for each of them, excluding the items where participants scored 0.

Regarding the IPAQ-SF, prior to the calculation of energy expenditure (MET-minutes/week) and the categorization of subjects, raw data were cleaned strictly following the official protocol guidelines (IPAQ Research Committee, 2005). Specifically, the exclusion rule was applied to activity episodes with a duration of less than 10 continuous minutes (recoding them to zero), and the truncation rule was applied to prevent overestimation due to outliers, limiting time variables (walking, moderate, or vigorous activity) that exceeded this threshold to a maximum of 180 min per day.

For data analysis, metabolic equivalents (MET-min/week) were calculated by multiplying the reported minutes and days by specific intensity coefficients: 3.3 METs for walking, 4.0 METs for moderate activity, and 8.0 METs for vigorous activity. Finally, total physical activity was calculated by adding together vigorous physical activity, moderate physical activity and the physical activity derived from walking.

#### 2.4.1 Self-Organizing Maps (SOM)

To identify profiles among the participants, a person-centered approach was employed using SOM. In practical terms, this technique acts as a “smart map” that visually organizes the data, generally placing students with similar behavioral and psychological characteristics close to one another on a two-dimensional grid while separating those with more dissimilar profiles. Unlike conventional clustering techniques, SOM provides a topology-preserving low-dimensional representation of multidimensional survey data, facilitating the visualization and exploration of complex patterns that may be difficult to identify using distance-based clustering methods alone. This unsupervised artificial neural network algorithm is particularly advantageous for handling complex, non-linear data structures without assuming a normal distribution. All computational procedures were performed using MATLAB R2021b (MathWorks Inc., Natick, MA, USA) and the SOM toolbox (version 2.0) for MATLAB. Prior to training the neural network, all continuous input variables included in this analysis (age, FAS, RIA, PIA, frequency of AI use, use of AI as a psychological assistant, total physical activity, connectedness, civil participation, positive social comparison, authentic self-presentation, and self-control) were normalized to a range of [0, 1] using a linear operation. Regarding missing data, no prior imputation or case deletion was required, as the SOM algorithm is inherently robust to incomplete datasets.

The SOM protocol followed a rigorous three-stage methodology. Initially, the neural network was constructed by defining a two-dimensional lattice size of 8 × 13 (width × height) neurons, which was determined based on the total sample size of the study (Bachner et al., 2022). Subsequently, the initialization phase was carried out to assign starting weights to each neuron across all input variables using two distinct approaches: randomized and linear initialization. Finally, the network underwent a training phase utilizing both sequential and batch algorithms (Oliver et al., 2018).

During the iterative training process, each participant's input vector is introduced to the lattice. The network's neurons compete to match the input vector by calculating the minimum Euclidean distance between their own weight vector and the participant's input vector. The “winning” neuron, along with its topological neighbors, adapts its weight values to more closely resemble the input vector (Molina-García et al., 2018). The magnitude of this adaptation is regulated by a decreasing learning rate and a neighborhood function, ensuring that the winning neuron adapts the most while the effect gradually decays for more distant neurons on the grid.

To control for the stochastic nature of the algorithm, such as the initial weight assignment and the entry order of the vectors, the entire training procedure was iterated 100 times. Consequently, a total of 1,600 independent SOMs were generated resulting from the combination of two initialization methods, two training algorithms, and four distinct neighborhood functions (100 × 2 × 2 × 4). The optimal map was then selected by first identifying the structural solutions that achieved a topographical error of zero, which guarantees that the spatial structure and neighborhood relationships of the input data are preserved intact. From this subset, the map yielding the lowest quantization error was retained, ensuring representation accuracy. Following the identification of the best SOM structure, a k-means algorithm was applied to group the 200 output neurons according to their connection weights. We opted for this two-stage hybrid strategy because relying solely on a single-stage unsupervised network frequently generates an extensive set of topological nodes that complicates quantitative analysis and practical interpretation. Implementing the k-means technique on the SOM outputs successfully minimizes data complexity and filters out potential noise. It achieves this by aggregating exceptionally stable pre-processed neural prototypes, acting as local averages, rather than processing the raw and potentially noisy individual data points. Consequently, this methodological design naturally overcomes common initialization and stability challenges, providing better boundary definition and classification accuracy than direct clustering approaches (Chang and Chen, 2015). The optimal number of final clusters was determined by testing solutions ranging from 2 to 10, selecting the definitive configuration based on the lowest Davies-Bouldin index, which indicates the best balance between within-cluster similarity and between-cluster separation. These emergent clusters served to characterize the specific behavioral and psychological profiles of the first-year university students regarding their sociodemographic variables, digital flourishing, physical activity levels, and perception of AI.

### 2.5 Statistical analysis

Following the SOM analysis, the assumptions of normality and homoscedasticity were assessed using the Shapiro-Wilk and Levene's tests, respectively for the variables related to emotional intelligence (Attention to feelings, Emotional clarity, and Emotional repair). Although the assumption of normality was not strictly met for some variables (Attention to feelings), parametric tests were maintained. According to the Central Limit Theorem, ANOVA is highly robust to deviations from normality when the sample size per group is sufficiently large (*n* ≥ 30) (Field, 2018). Given that the smallest cluster in our study comprised approximately 50 participants, and the assumption of homoscedasticity was confirmed across all variables via Levene's test (*p* > 0.05), the use of a one-way ANOVA was deemed statistically appropriate and robust against non-normal distributions (Blanca et al., 2017).

Therefore, a one-way ANOVA was conducted to compare the values of these variables across the different profiles identified by the SOM clusters. When significant main effects were found, Tukey's *post-hoc* tests were performed for pairwise comparisons. The significance level for all statistical analyses was set at *p* < 0.05.

## 3 Results

**Figure 1:**
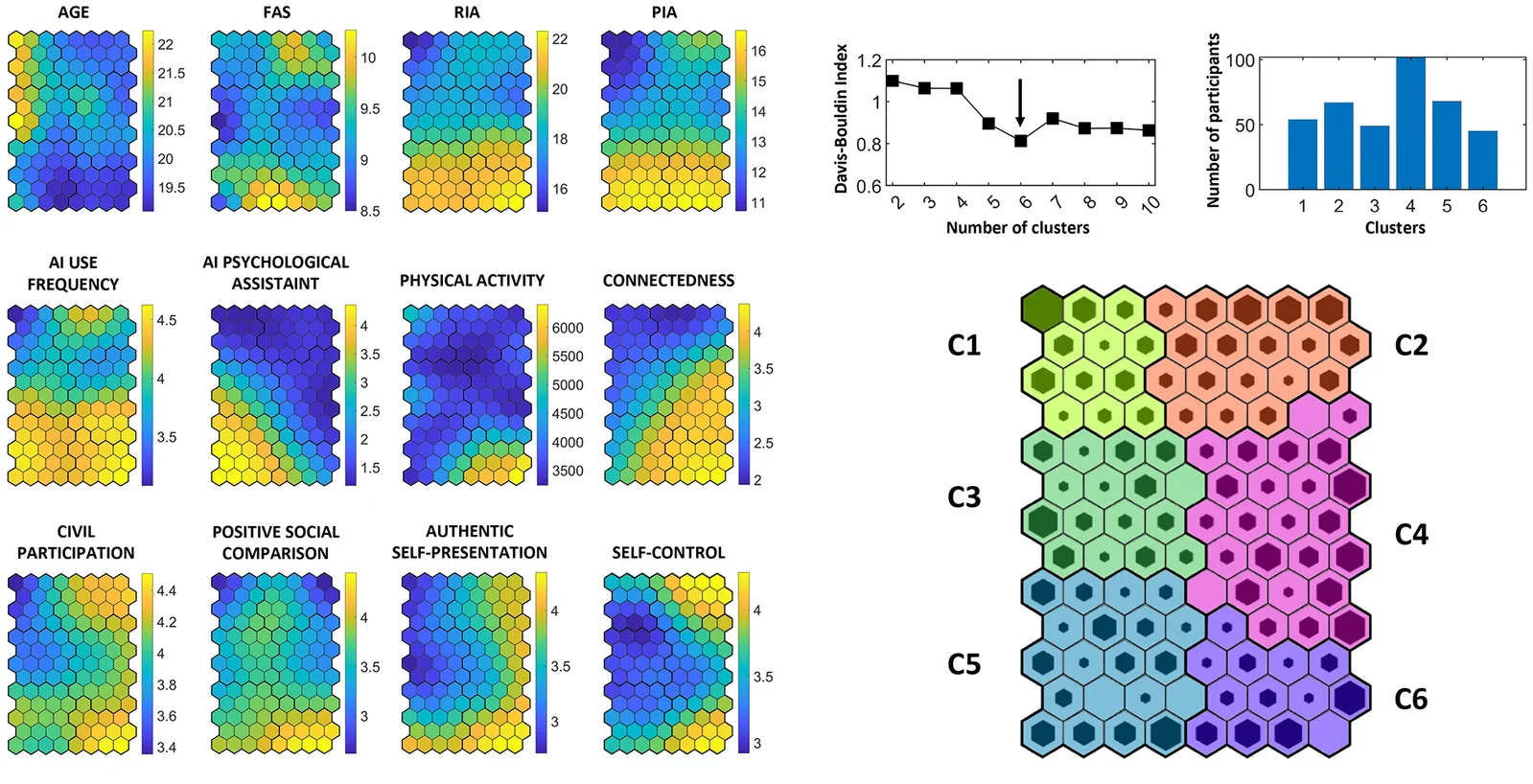
Self-Organizing Map visualization of the six differentiated student profiles. The component planes illustrate the distribution of each variable across the neural network. Warmer colors (yellow/green) indicate higher values, whereas cooler colors (dark blue/cyan) represent lower values relative to the sample distribution. The top-right section displays the cluster selection plot based on the Davies-Bouldin index and the distribution of subjects per cluster, respectively. Finally, the bottom-right map displays the final topological clustering of the six identified profiles.

The first objective of this study was to identify distinct topological profiles among the participants based on their sociodemographic variables, physical activity levels, digital flourishing, and AI adoption patterns. To achieve this, a SOM neural network was trained using the standardized input variables. The Davies-Bouldin index indicated that a six-cluster solution provided the most robust and differentiated grouping (Figure 1).

Visual inspection of the component planes reveals distinct aggregation patterns across the neural network, with warmer colors representing higher relative values and cooler colors indicating lower relative values within the sample. A detailed analysis of these maps highlights a strong contrast between two specific regions of the network.

On the bottom-right region, C6 emerged as a highly active and digitally integrated profile. The component planes for this cluster display a high concentration of warm colors in the Physical Activity dimension and across all Digital Flourishing variables (Connectedness, Civil Participation, Positive Social Comparison, Authentic Self-Presentation, and Self-Control). Furthermore, this group exhibited the highest frequency of AI use, alongside prominently high scores in their positive engagement and enthusiasm toward AI-assisted learning (RIA), as well as perceived AI accuracy (PIA). Concurrently, this profile displayed moderate-to-cool tones in the utilization of AI as a psychological assistant.

Conversely, the top-left region of the network, corresponding to C1, displayed a diametrically opposed pattern. This cluster was predominantly characterized by cool colors across almost all dimensions, indicating the lowest relative levels of physical activity, profound deficits in digital flourishing, and minimal scores in positive engagement (RIA), perceived accuracy (PIA), and general adoption of AI tools. The remaining clusters (C2, C3, C4, and C5) occupied intermediate topological positions, displaying mixed configurations of behavioral and digital habits.

Based on these topological distributions, C6 and C1 were conceptualized as the adaptive and vulnerable profiles, respectively, representing the two extremes of the behavioral and technological adaptation spectrum in our cohort.

To address the second objective of the study, a one-way ANOVA was conducted to determine whether there were significant differences in the dimensions of intrapersonal emotional intelligence (attention, clarity, and repair) across the six topological profiles identified by the SOM. The analysis revealed statistically significant main effects for all three emotional dimensions: Attention to feelings [*F*_(5, 379)_ = 3.56, *p* = 0.004, η^2^ = 0.04], Emotional clarity [*F*_(5, 379)_ = 5.33, *p* < 0.001, η^2^ = 0.07], and Emotional repair [*F*_(5, 379)_ = 4.21, *p* < 0.001, η^2^ = 0.05]. Subsequently, Tukey's *post-hoc* tests for multiple comparisons were performed to specify the differences between the clusters (see Table 1).

**Table 1:** Differences in emotional intelligence dimensions across the six SOM profiles.

Cluster	Attention to feelings	Emotional clarity	Emotional repair
1	25.9^†^^*^ (7.05)	23.9^*^ (7.26)	24.5^*^ (6.18)
2	28.6 (5.78)	26.3 (6.41)	26.6 (6.08)
3	27 (6.61)	23.9^*^ (6.45)	24.5^*^ (5.20)
4	27.9 (6.17)	25.5^*^ (6.02)	25.3^*^ (6.63)
5	29.2 (6.46)	26^*^ (7.22)	26.2 (7.05)
6	30.6 (6.22)	30 (7.67)	29.5 (6.29)

Data are presented as Mean (Standard Deviation). Statistically significant differences according to Tukey's *post-hoc* test are denoted by the following symbols:^†^Indicates significant differences compared to Cluster 5 (*p* < 0.05); ^*^Indicates significant differences compared to Cluster 6 (*p* < 0.05).

## 4 Discussion

The primary objective of this study was to explore the existence of distinct behavioral and psychosocial profiles among incoming university students based on their sociodemographic variables, lifestyle, digital flourishing, and AI adoption. Utilizing SOM, the analysis identified six differentiated profiles, suggesting that these behavioral and technological dimensions do not merely operate in isolation but may aggregate into specific typologies during the university transition. Notably, subsequent inferential analyses and pairwise comparisons revealed that the most pronounced differences emerged in the regulatory dimensions of intrapersonal emotional intelligence. Specifically, Cluster 6 exhibited the most favorable scores, significantly outperforming Clusters 1, 3, 4, and 5 in emotional clarity, as well as Clusters 1, 3, and 4 in emotional repair. In contrast, the dimension of emotional attention displayed a more homogeneous distribution across the remaining typologies, although Cluster 1 still presented significantly lower scores compared to the most adaptive profiles. Consequently, while the other identified typologies highlight the complex and nuanced nature of student adaptation, the marked difference across these regulatory competencies solidifies the conceptual definition of these opposing extremes as the adaptive profile (Cluster 6) and the vulnerable profile (Cluster 1), representing opposite ends of the behavioral and psychological adjustment spectrum within the analyzed cohort.

The characteristics observed in the adaptive profile tend to support the notion of a positive synergy between active physical habits and digital wellbeing (Pirwani and Szabo, 2024). Specifically regarding technological engagement, students within this profile appear capable of maintaining a high frequency of AI integration for instrumental and academic purposes (accompanied by a positive affective receptivity) while simultaneously exhibiting moderate-to-low tendencies to utilize AI as a psychological assistant. This balanced approach to AI might be closely linked to their higher capacities for emotional clarity and repair. The possession of robust internal psychological resources to effectively process and regulate emotional states is associated with a more balanced and effective adoption of automated systems in these students (Suriá-Martínez et al., 2025). Within the specific context of our cohort, this balanced adoption appears to translate into a self-sufficient approach; having robust internal regulation, these students may not feel the need to rely on external AI tools to manage transition-related anxieties or act as a psychological assistant.

Conversely, the vulnerable profile (Cluster 1) highlights a pattern where low relative physical activity and limited digital flourishing coincide with lower emotional competencies. Given that physical activity can positively influence life satisfaction and psychological wellbeing through the mediating role of emotion regulation mechanisms (Nie et al., 2025), the relative inactivity observed in this cluster is likely linked to a reduced utilization of this valuable regulatory resource. Rather than indicating a permanent deficit, this trend might reflect a state of transient vulnerability exacerbated by the highly demanding nature of the university transition. Lower levels of intrapersonal regulatory resources (particularly emotional clarity and repair) are often associated with greater difficulties in coping with the diverse stressors associated with this transitional stage (Thomas and Zolkoski, 2020; Mastrokoukou et al., 2024). Consequently, this vulnerability in psychological buffering is observed alongside lower digital flourishing and digital emotional intelligence, a context that may be less conducive to the cultivation of positive connections and the maintenance of healthy digital self-control (Audrin and Audrin, 2023). Instead of thriving within the university's digital ecosystem, these students might resort to technological avoidance or experience reduced digital self-efficacy as a maladaptive coping mechanism to manage technological demands (Asad et al., 2023).

Consequently, the findings of this study suggest that the goal of higher education institutions might transcend the mere restriction or distancing of students from AI and emerging technologies, creating instead new policies and scenarios for their proper integration into the university ecosystem (Sun et al., 2026). Therefore, moving beyond purely preventive approaches, the efforts of academic counseling and tutoring departments could focus on equipping students with the necessary tools and competencies to promote an instrumental, critical, and healthy use that fosters their digital flourishing. In this scenario, adequate levels of intrapersonal emotional intelligence could play a crucial facilitating role. By acting as an internal self-regulatory resource, higher levels of emotional clarity and repair could be related to a better management of the negative factors associated with hyperconnectivity (Shaban et al., 2025), and aligned with a more conscious and autonomous technological adoption, enabling them to harness the potential of AI while safeguarding their psychological wellbeing (Suriá-Martínez et al., 2025).

On the other hand, the adaptive profile (Cluster 6) offers a valuable perspective for guiding these initiatives. In this group, the combination of active lifestyles and high emotional competencies suggests the utility of implementing holistic institutional programs that go beyond conventional technological literacy. Consequently, student counseling and welfare services could integrate intrapersonal emotional regulation workshops alongside the promotion of physical exercise. This approach is supported by recent evidence indicating that physical activity is positively related to mental health in university cohorts, particularly when accompanied by adequate emotional regulation and self-efficacy (Wang et al., 2025). By addressing both aspects, institutions could strengthen students' digital resilience, helping them navigate an increasingly technological academic environment more effectively.

Despite the methodological and theoretical contributions of this study, several limitations must be acknowledged to properly contextualize the findings. First, the cross-sectional nature of the research design precludes the establishment of definitive causal relationships between lifestyle behaviors, digital flourishing, and emotional intelligence. While the SOM analysis identified clear concurrent typologies, the directionality remains tentative; it is unclear whether robust emotional regulation facilitates a more active lifestyle or if physical engagement actively drives psychological adaptability during the first-year transition. Second, the reliance on self-reported instruments introduces potential subjectivity bias, as is often the case with physical activity questionnaires, where participants frequently tend to overestimate their actual activity levels. Similarly, regarding emotional intelligence, while a self-report approach was chosen for its feasibility within a comprehensive test battery, these measures assess perceived efficacy rather than objective capability. Therefore, future research would benefit from incorporating objective maximal-performance tests, such as the MSCEIT (Extremera et al., 2006), to complement these findings. Furthermore, while this study focused strictly on intrapersonal factors, future lines of inquiry should broaden this scope to include interpersonal emotional dimensions (e.g., using the WLEIS, Sanchez-Garcia et al., 2016) to understand the relationship between social emotional skills, digital flourishing, and AI adoption. Finally, the sample was restricted to first-year students within a specific institutional framework and presented a significant sex imbalance (298 females, 87 males). This disproportion precluded robust inferential comparisons between sexes regarding emotional intelligence dimensions. As a major limitation, this demographic and institutional homogeneity means that the identified profiles should be interpreted with caution, as they may not fully capture the behavioral and psychological realities of male students, advanced undergraduates, or populations from different geographical and sociocultural backgrounds. Therefore, the broader generalizability of these specific topological clusters is limited. Future longitudinal and experimental designs are warranted to elucidate the causal trajectories of these relationships and to test the efficacy of integrated, multi-domain interventions within the higher education ecosystem.

In conclusion, this study utilized a person-centered topological approach (i.e., SOM) to identify six distinct behavioral and psychosocial profiles among first-year university students. The findings show a clear difference between an adaptive profile (Cluster 6, characterized by an active lifestyle, high digital flourishing, and an instrumental use of AI) and a vulnerable profile (Cluster 1, marked by relative physical inactivity and lower levels in the digital dimensions). Furthermore, subsequent inferential analysis confirms that these profiles exhibit significant differences in their levels of intrapersonal emotional attention, clarity, and repair. Collectively, these insights suggest that successful adaptation to the university and technological environment transcends the mere acquisition of isolated skills, underscoring the potential benefit of institutional strategies that simultaneously integrate the promotion of physical activity, digital wellbeing, and emotional competence.

## Data Availability

The raw data supporting the conclusions of this article will be made available by the authors, without undue reservation.
